# Friction characteristics of Cd-rich carbonate films on calcite surfaces: implications for compositional differentiation at the nanometer scale

**DOI:** 10.1186/1467-4866-10-7

**Published:** 2009-06-23

**Authors:** Pablo Cubillas, Steven R Higgins

**Affiliations:** 1 Department of Chemistry, Wright State University, 3640 Col. Glenn Hwy. 45435 Dayton, Ohio, USA; 2 Centre for Nanoporous Materials, School of Chemistry, The University of Manchester. Oxford Road M13 9LP, Manchester, UK

## Abstract

Lateral Force Microscopy (LFM) studies were carried out on cleaved calcite sections in contact with solutions supersaturated with respect to otavite (CdCO_3_) or calcite-otavite solid solutions (SS) as a means to examine the potential for future application of LFM as a nanometer-scale mineral surface composition mapping technique. Layer-by-layer growth of surface films took place either by step advancement or by a surface nucleation and step advancement mechanisms. Friction vs. applied load data acquired on the films and the calcite substrate were successfully fitted to the Johnson Kendall Roberts (JKR) model for single asperity contacts. Following this model, friction differences between film and substrate at low loads were dictated by differences in adhesion, whereas at higher load they reflect differences in contact shear strength. In most experiments at fixed load, the film showed higher friction than the calcite surface, but the friction-load dependence for the different surfaces revealed that at low loads (0–40 nN), a calcian otavite film has lower friction than calcite; a result that is contrary to earlier LFM reports of the same system. Multilayer films of calcian-otavite displayed increasing friction with film thickness, consistent with the expectation that the film surface composition will become increasingly Cd-rich with increasing thickness. Both load- and thickness-dependence trends support the hypothesis that the contact shear strength correlates with the hydration enthalpy of the surface ions, thereby imparting friction sensitivity in the LFM to mineral-water interface composition.

## Introduction

The study of ion sorption onto mineral surfaces has received considerable attention in the last several decades. Sorption is, in the broad sense, the change of mass of a chemical in the solid phase as a result of mass-transfer between fluid and solid, which includes, 1) true adsorption (chemical or electrostatic), 2) absorption or diffusion into the solid, and 3) surface precipitation to form an adherent phase that may consist of chemical species derived from both the aqueous solution and dissolution of the solid [1]. Thus sorption plays an important role in processes such as the cycling of elements, diagenesis, and the removal of heavy metals from contaminated waters. Furthermore, the interaction of ions with mineral surfaces can influence the crystallization behavior of secondary minerals, affecting the formation of polymorphs, crystal morphology, and inhibiting nucleation and growth as well as dissolution. In this context the study of the interactions between carbonate minerals and metal ions has been the topic of numerous investigations [2-8] since these minerals are some of the most ubiquitous rock forming minerals in the Earth and they are often present in aquifers as well as in sediments and soils [9]. Biogenic calcium carbonates are also very common and the incorporation of elements such as Mg and Sr has been related to particular formation parameters such as temperature, water chemistry, and nutrient levels [10].

When dealing with mineral-ion interactions, it is important to consider both the saturation states of possible precipitating solids with end-member composition and of the solid-solutions. Certain solid-solution compositions can theoretically form even when their end members remain undersaturated [7,11]. This is especially true when dealing with interactions between calcite and Cd^2+^(aq), since this ion is known to substitute for Ca^2+ ^in the calcite structure [12]. Past studies have focused on understanding ion partitioning and other fundamental aspects of the ion-carbonate surface interaction [13,14] whereas more recent work has focused on determining the sorption capacity of carbonates and its environmental implications [7,15,16].

The interactions between carbonates and heavy metals have been studied in the past using a variety of experimental techniques. These techniques range from traditional "macroscopic" methods such as batch and flow-through reactors [7,15,17], to surface sensitive techniques [13,18-22] to studies at the "nanoscale" performed by means of Atomic Force Microscopy [11,23-29].

AFM investigations have proved to be extremely valuable in expanding the degree of knowledge of mineral-ion interactions. They have provided surface topographic evidence in evaluating crystal nucleation and growth mechanisms as well as on the inhibitory effect of ions on growth or dissolution processes. Nevertheless, few studies have gone further in the use of modified scanning modes of the AFM, such as Lateral Force Microscopy (LFM). LFM studies have been carried out mainly in the fields of nanotribology [30-34] and material sciences [35] where investigations were directed toward understanding the origin of contact mechanical properties such as friction, adhesion, wear, indentation, and thin-film lubrication. In contrast, LFM studies in systems of geochemical interest were scarce up until recently [29,36-44].

One of the aims of the present work was to study and quantify lateral forces produced at the AFM tip-mineral interface by growing (Cd, Ca)CO_3 _solid solutions on calcite surfaces. This system was previously studied by Hay et al. [29] using LFM; nevertheless these authors did not provide quantitative data on the friction differences between calcite and the mixed cation phases although it is realistic to assume that lateral forces and surface composition are related. For example, in a study of (Ca, Mg)CO_3 _film growth on dolomite, higher friction was generally observed on Ca-rich films (Ca/Mg ratio > 1) [44] in comparison with dolomite, whereas in Mg-rich films, little if any difference in friction was reported [40]. Higgins et al. [44] showed that the hydration enthalpy of the ions in dolomite led to predicted friction forces that are in the same range as those forces observed experimentally, but in their study, significant film strain complicated the interpretations of the quantitative friction data. In the present work, experiments were designed to test the hypothesis that the hydration enthalpy of the ions exposed at the mineral-water interface play an important role in the friction characteristics of the LFM probe-surface contact. By studying Cd^2+ ^as the dopant for the formation of Cd-rich surface films, film strain and its effects on the mechanical properties of the LFM probe-surface contact should be relatively small owing to the small lattice mismatch between the surface unit meshes on otavite and calcite. To this end, experimental investigations were conducted using the LFM to map differences in friction (as a function of applied load) observed on surface domains of Cd-enriched carbonate films grown on calcite (10^-4^) surfaces.

## Methods

### Friction Force Microscopy and continuum mechanics

**Figure 1:**
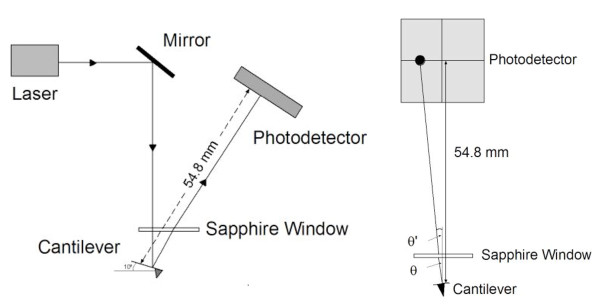
**Simplified diagram of the photodetector set-up and laser path**. The diagram shows the lateral view (a) and front view (b) of the laser path and photodector set up. The original angle formed by the reflected beam on the cantilever (2*θ*) changes to 2*θ*' from refraction by the sapphire window (2*θ*' = 2*θ*/1.33).

An Atomic Force Microscope measures the forces between a sharp tip (with a typical radius of 10–100 nm) and a sample surface. Most commonly, the tip is mounted on the end of a cantilever that serves as a force sensor. Variations in the sample-tip force results in the deflection of the cantilever, which can be monitored by the change in position of a laser beam reflected from the cantilever surface into a four-quadrant photodiode. During the scanning process the cantilever will bend due to forces acting normal to the sample surface. In addition, the cantilever will twist due to lateral (friction) forces between the tip and the substrate that opposes relative movement between the two. This twisting is detected by the photodiode mechanism as a change in the "lateral" position of the laser spot (Fig. 1). In the monitoring of these "lateral forces" by an AFM, in the daughter method of LFM, the lateral force signal measured for forward or backward scans will have opposite sign since the cantilever will twist in opposite directions. A plotting of the lateral force signal vs. lateral displacement for both scans defines the so-called friction loop. Friction force, then, is defined as the half of the difference between the two signals measured in the opposing scanning directions [44,45].

The tip-sample contact constitutes a single asperity contact. In these cases it has been shown that friction has a non-linear dependence with the load applied [46], contrary to the linear dependence exhibited for multi-asperity contacts (Amonton's Law, 1698). The AFM tip-sample contact has been successfully described by means of continuum contact mechanics models [33,47-49]. The original model was proposed by Hertz [50], and assumes that no attractive forces act between the two materials; nevertheless experiments have shown the existence of adhesion forces between them. Two models were developed afterwards that take this fact into account. The Johnson-Kendall-Roberts (JKR) model [51] and the Derjaguin, Müller and Toporov (DMT) model [52]. The former is valid, and assumed to be valid in these investigations, when dealing with relatively soft materials and short range adhesive forces, whereas the latter describes better the interactions between harder materials. The description of contacts that lie within these two extremes are addressed by considering a transition parameter know as the Tabor parameter [49,53].

The JKR model, originally developed to address the case of two spheres in contact, is extended to a plane-sphere contact by considering an infinite radius for one of the spheres. This theory, as well as the Hertz and DMT models, predicts a load (***L***) dependency of the contact area (***A***) between the sphere and the plane, which, for the JKR model is defined as:(1)

where *R *is the tip radius, *γ *is the interface surface energy or Dupré energy of adhesion, which corresponds to the work per unit area required to separate the surfaces from contact to infinity, and *K *is the reduced elastic modulus of the two materials in contact and is defined by [eg., [54]]:(2)

where *E*_1 _and *E*_2 _are the Young's Moduli and *υ*_1 _and *υ*_2 _are the Poisson ratios for the sphere and plane materials. The JKR theory predicts that a finite negative or critical load (***L***_***c***_) is required to separate the surfaces, and is given by:(3)

This is equivalent to the pull-off force measured in the AFM. At the critical load, therefore, a finite contact area will exist.

Friction *F*_***f ***_force has been shown to be proportional to the surface area in experiments performed with the surface forces apparatus (SFA) [55]:(4)

where *τ*_0 _represents the shear strength of the contact. Proportionality between friction and contact area has been observed for elastic, wearless, single asperity contacts. Nevertheless, the shear strength could also be a function of applied load, as suggested by Sørensen et al. [56] in their theoretical studies, and corroborated by Briscoe and Evans [57] on boundary lubrication experiments with monolayers of fatty acids between molecularly smooth mica surfaces.

**Figure 2:**
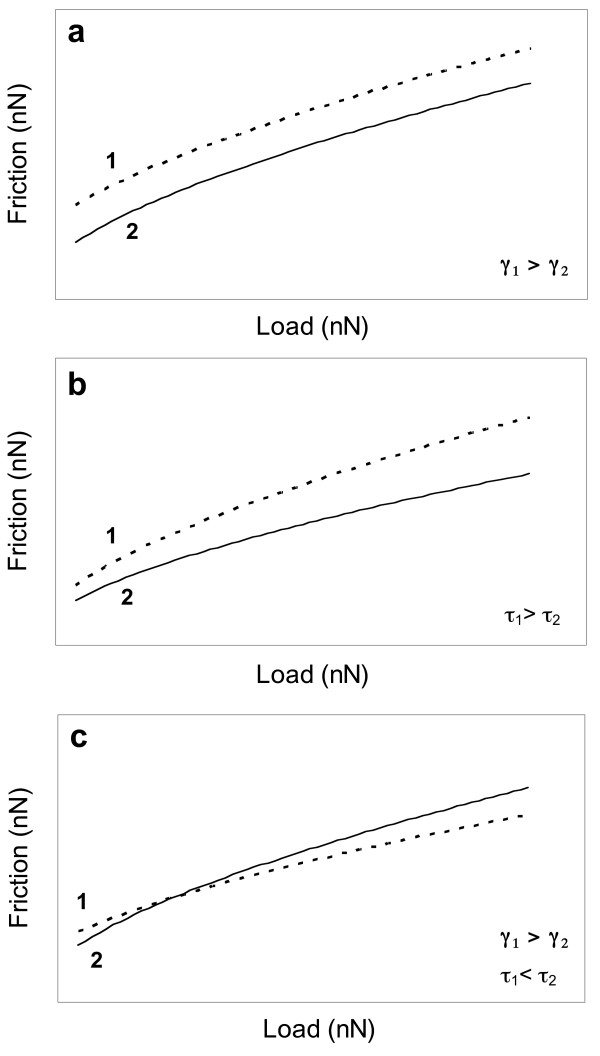
**Theoretical friction vs load curves for two different materials in contact with an AFM tip**. a) Contact 1 has a higher surface adhesion than contact 2 (*γ*_1 _> *γ*_2_) but the same shear strength. b) Shear strength of contact 1 is higher than that of contact 2. c) Shear strength of contact 1 is lower than contact 2; adhesion of contact 1 is higher than contact 2.

By combining equations 1, 2 and 4 it is possible to model the load dependence of friction for a contact between an AFM tip and a surface. Fig. 2 shows different theoretical friction vs load curves for two different tip-sample contacts highlighting the effect of the contact shear strength (*τ*) and adhesion energy (*γ*). Fig. 2a shows the theoretical friction vs load curves for two substrates with different adhesion energies but the same contact shear strength. This difference does not affect the overall slope of the curves. In Fig. 2b the shear strength of contact 1 is higher than that of contact 2 but the adhesion energies are the same. This translates into a different slope for each curve. In fig. 2c both the contact shear strength and the adhesion energies are different for the two materials. In this case both curves will intersect each other at a certain point.

### Experimental set up

Experiments were performed using a custom-built AFM [58] equipped with a flow cell. This flow cell was designed to produce a vertically impinging solution jet onto the sample surface within 2 mm of the tip contact [59]. All experiments were performed in contact mode using silicon cantilevers (Point Probe Plus-CONT). In these investigations, cantilever load was varied to compare the load-dependent lateral force against a mechanical model for sphere-plane contacts. Load was calculated with respect to the free position of the cantilever. Calcite samples were freshly cleaved along the (10^-4^) plane in air from optically clear Iceland Spar crystals (Ward's Natural Science Est. Inc.). Typical dimensions of the cleaved crystals were around 5 mm × 5 mm × 1 mm. Samples were introduced into the fluid cell immediately after cleavage to prevent contamination. Prior to the start of the experiment a calcite-undersaturated solution of CaCl_2 _(0.3 mmol/L) and NaHCO_3 _(0.3 mmol/L) (pH = 7.5 – 8) was flushed through the cell to promote slow dissolution of the calcite sample and to generate etch pits. Afterwards, a solution containing the desired ion combinations was introduced. The total duration of the experiments varied between 2 and 20 hours. Fluid flow rate was maintained constant to 2.5 ± 0.1 g/hr using a Porter Instrument mass flowmeter and flow controller.

Solutions were prepared using de-ionized water (18 MΩ cm resistivity), and high-purity CaCl_2_, NaHCO_3_, CdCl_2_, and NaOH reagents. pH adjustment of the inlet solutions was achieved by adding small amounts of a NaOH solution (0.1 mol/L) to the initial solution. Once the desired pH was achieved the solution was injected into the flow system by means of a Luer-Lok^® ^20 ml syringe. Re-equilibration kinetics of these solutions with CO_2 _under the pH ranges used (8–9.5) were slow, so the pH measured just before introducing the solution into the CO_2 _free flow system was considered to be the pH of the solution in the flow cell where the calcite sample was located. pH measurements were carried out using an Accumet^® ^pH/ATC Combination electrode (Fisher Scientific 1961/Accumet) connected to a dual ion/pH meter from Fisher Scientific. The pH electrode was calibrated using pH 4, 7, and 10 buffer solutions from Fisher Scientific.

Activities and saturation indexes of the solutions were calculated using the program PHREEQC [60] and the database Phreeqc. Saturation index is defined as:(5)

where *IAP *stands for the ion activity product and ***K***_***SP ***_for the solubility product of the solid phase. The saturation state of a solid solution (B, C)A is not represented by a single value but is a function of both the solid and aqueous phase compositions. The general expression of the supersaturation function has the form [61]:(6)

where x is the solid composition, [***A***^-^] refers to the activity of ion A in the aqueous solution, *K*_*BA *_refers to the solubility product of the *BA *end-member and *X*_*BA *_is the mole fraction and *γ *the activity coefficients. The maximum of the supersaturation function provides a good approximation of the precipitating solid-solution composition; nevertheless it does not take into account the kinetics of the nucleation process. More accurate predictions of the composition of the precipitating solid-solution requires this latter consideration (with the knowledge of a number of experimental parameters) as has been shown by several authors [11,61,62]. The saturation states for the different solid solution compositions were calculated using the supersaturation function defined by [61]. This function was added into Phreeqc as BASIC programming code.

### Calibration of the LFM signal

The magnitude of the friction force, ***F***_***f***_, in an atomic force microscope can be calculated by [63]:(7)

where ***V***_***L ***_is the difference in the lateral force signal detected (V), ***S***_***L ***_is the lateral detector sensitivity (rad/V), ***K***_***L ***_is the torsional spring constant (nN m/rad), and ***h ***is the tip length of the cantilever (≈ 15 *μ*m).

The sensitivity of the detector as a function of the angle of twisting of the cantilever was calculated by accounting for the specific geometry of the laser optical system and detector. Using the four-quadrant photodiode detector sensitivity along its horizontal axis (9.26 *μ*m/V), it was possible to determine the relative laser spot shift (in *μ*m) for a given voltage difference, *V*_*L*_, measured from friction loops [e.g., [44]]. By considering the distance of the laser spot on the cantilever to the detector and taking into account the change of path of the laser due to refraction when going through the water/sapphire window and sapphire window/air interfaces (Fig. 1), it was possible to determine the angle of twist (*θ*) of the cantilever for a given voltage. This calculation yielded a detector sensitivity of 1.26 × 10^-4 ^rad/V.

The torsional spring constant (*K*_*L*_) is related to the normal spring constant by the following expression [64]:(8)

where *K*_***N ***_is the normal spring constant (N/m), *L *is the length of the cantilever (450 *μ*m nominal) and *υ *is the Poisson ratio (0.28 for Si). The normal spring constant for each cantilever utilized was determined experimentally using a calibrated cantilever as a reference and the method described by [65]. Therefore, by measuring the *K*_***N ***_of every cantilever, it was also possible to calculate its torsional spring constant and calibrate the friction forces in the LFM measurements using Eqn. 7 and 8.

### Friction force measurements

Measured friction can be influenced by different variables and experimental factors, such as the tip sliding velocity [66-68], the position of the laser on the cantilever [69], lateral spring constant determination and tip radius. In the current study all comparative data was taken at a fixed sliding speed and laser position so these two factors were not expected to be a problem.

The shape of the tip-sample contact has a significant influence on the friction force measured as evidenced by the continuum mechanics models discussed previously. Unfortunately tip shape can vary significantly between different tips, leading to different contact areas and/or contact geometries under otherwise invariant conditions. Also, the tip shape will be altered by the wear resulting from scanning over the sample. Several authors have dealt with these issues in the past [32,47,54,70-72] and have tried to determine the variation of the tip shape produced during the experiments for tips of different compositions. In this study, only Si cantilevers were used and they were imaged prior to and after the performance of the experiments. Imaging was performed by scanning a calibration grid of regularly spaced sharp asperities (MicroMasch, Inc.) under similar load conditions. Several experiments were run with the same tip, in order to address the issue of wear and its possible influence in the geometry and size of the contact area. Results from these experiments showed a non-uniform evolution of the tip shape, and they also revealed a significant elongation of the tip in the direction perpendicular to the scanning direction. This led to a change in the form of the tip apex, evolving from a sphere to a barrel shape. This will mean that the form of the tip-sample contact will evolve from a circle to an ellipse, with its long axis perpendicular to the scan direction. Absolute friction values showed no relation with the evolution of the tip shape, however, suggesting that other factors may have had more significant influences on the measured friction.

For the reasons stated above the direct comparison between calculated friction values even for experiments performed in separate sessions, yet at similar conditions, could be highly misleading and thus has been avoided in the discussion. Unfortunately, some of the experiments performed in this study showed that, even within an experiment, absolute friction values could vary up to 60%. Nevertheless, in this case the variation affected the friction values of the different layers in the same manner, making comparisons within experiments still possible. For those experiments where friction vs. load data was taken, a rapid change in the tip shape could have important implications on the data interpretation. In these cases, however, no significant variation (>20%) of the tip shape was observed before and after the experiment.

### Data analysis

A custom Matlab^® ^code was written to batch process and analyze the lateral force data obtained from the AFM experiments. Friction values were computed by dividing by two the difference in lateral deflection measured by a left-to-right scan and a right-to-left scan. Commonly, the scans do not overlap due to scanner hysteresis (i.e. features observed in forward and backward scans do not correspond to the same pixel position in the fast-scan axis), so the code includes a "matching" subroutine (based on lateral deflection image cross-correlation) that automatically overlaps both scans before calculating the friction. Several other features were included in the code for "region of interest" (ROI) statistical analysis in the images to obtain friction values and other statistical parameters or for automated analysis of "friction loops" [73,74].

## Results

To assess the friction of otavite-calcite solid solutions forming on calcite, a series of experiments were performed in which the inlet solution contained Ca^2+^(aq), Cd^2+^(aq) and CO_3_^2-^(aq) in different proportions, including Ca-free solutions. Table 1 shows the different solution compositions for all experiments performed plus the saturation index for otavite and calcite as well as the maximum of the supersaturation function and its corresponding solid solution (SS) composition.

**Table 1:** Experimental data summary. Solution composition, pH, saturation indexes and predicted solid solution composition for the experiments performed with Ca^2+^(aq)-Cd^2+^(aq)-HCO^-^_3_(aq) solutions.

Experiment	**Cd**^**2+ **^(mmol/L)	**Ca**^**2+ **^(mmol/L)	**NaHCO3 **(mmol/L)	pH	**SI**_**ot**_	**SI**_**cal**_	**SI**_**max**_^**a**^	**X**_**Cd**_^**b**^
Cd.1	0.1	-	0.1	8.4	3.17	-	-	-
Cd.2	0.1	-	0.1	8.5	3.35	-	-	-
Cd.3	0.1	-	0.1	7.96	2.34	-	-	-
Cd.4	0.1	-	0.1	8.1	2.6	-	-	-
Cd.5	0.1	-	0.1	8.23	2.81	-	-	-
Cd.6	0.1	-	0.1	8.2	2.75	-	-	-
Cd.7	0.1	-	0.1	8.22	2.83	-	-	-
Cd.8	0.1	-	0.1	8.28	2.91	-	-	-
Cd.9	0.1	-	0.1	8.24	2.83	-	-	-
Cd.10	0.1	-	0.1	8.27	2.89	-	-	-
Cd.11	0.1	-	0.1	8.08	2.56	-	-	-
Cd.12	0.1	-	0.1	8.2	2.75	-	-	-
Cd.13	0.001	0.34	0.9	8.75	1.54	1.63	1.89	0.92
Cd.14	0.001	0.34	0.9	8.51	1.29	0.25	1.32	0.92
Cd.15	0.001	0.34	0.9	9	2.12	1.1	2.16	0.91
Cd.16	0.001	0.34	0.9	8.54	1.36	0.31	1.4	0.92

a. Maximum reached by supersaturation function.

b. Solid solution composition corresponding to the maximum of the supersaturation function.

### Film friction-load characteristics

**Figure 3:**
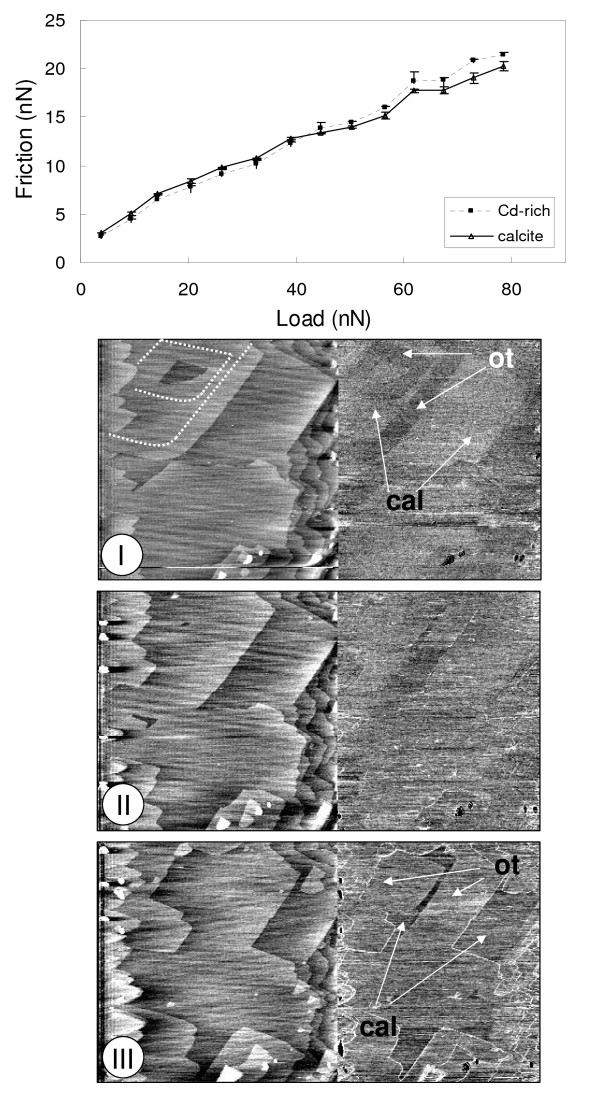
**Friction versus load plot for experiment Cd-1 and related height/fricion images**. a) The friction vs. load plot shows that at low loads the calcite contact possess a slightly higher friction than that calculated for a single layer of calcian otavite, but at higher loads, the friction force is larger on the calcian otavite film. b) Height/friction image corresponding to a load of 14 nN. White lines on the height image represent the original calcite steps prior to the start of the calcian otavite film growth. c) Height/friction images corresponding to a load of 40 nN. d) Height/friction images corresponding to a load of 72 nN.

As evidenced by the theoretical continuum mechanics models previously discussed, the friction characteristics of a tip-film contact can be dominated by different parameters at different load regimes (Fig. 2). To assess these parameters, a series of friction measurements as a function of load where carried out on calcite and Cd-rich films in experiments Cd.1 and Cd.11. Results from experiment Cd.1 are shown in Fig. 3. Fig. 3a shows the friction vs. load measurements for both the calcite and Cd-rich film. At low load (<40 nN) the friction for the calcite contact is higher than that of the Cd-rich film. Although small, the differences in friction are real as can be seen in Fig. 3b which shows the height and friction signals recorded at a load of 15 nN. At medium loads (≈40 nN) the measured friction for both contacts is approximately the same (Fig. 3c). At higher loads the friction contrast reverses and is the Cd-rich film contact the one displaying the higher friction. The recorded differences are again small, but significant (Fig. 3d). Results from experiment Cd.11 corroborate the differences in the load-dependent friction for the two contacts. In comparison, Hay et al. [29] only reported that the Cd-rich layers had a higher lateral force signal than the calcite substrate.

### Film growth from Ca^2+^(aq)-free inlet solutions

**Figure 4:**
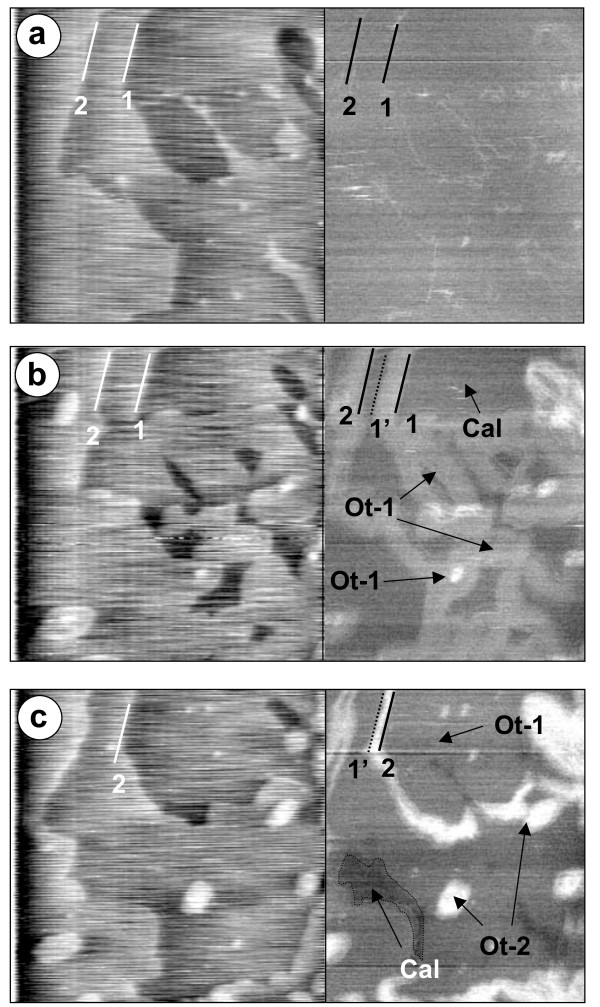
**AFM sequence of images for experiment Cd.6 performed at a load of 25 nN**. Each pair of images shows a topography image on the left and a friction image in the right, corresponding to the same scan. a) calcite surface before switching to the Cd^2+^(aq) rich solution. The position of steps 1 and 2 are highlighted in both images. b) Growth of a first layer of calcian otavite (Ot-1) as well as the formation of calcian otavite nuclei (Ot-2) over Ot-1, 50 min after the introduction of a CdCO_3 _supersaturated solution. The right image shows the contrast in friction between the two calcian otavite layers and the original calcite. Also clear is the initial position of step 1 prior to its advancement (labelled 1') c) Calcite surface nearly completely covered by a layer of calcian otavite (Ot-1) 145 minutes after introduction of supersaturated solution. The position of step 2 has advanced over the original position of step 1, forming a double layer of calcian otavite (Ot-2). The areas occupied by two layers of calcian otavite show a much higher friction signal than those where only one layer of calcian otavite has grown. Image size: 5 *μ*m × 5 *μ*m.

Fig. 4 shows topography and friction images taken with a load of 25 nN during experiment Cd.6 where a calcite crystal reacted with a Ca-free solution supersaturated with respect to Otavite (SI_**ot **_= 2.75). Five minutes after the solution was introduced into the cell, the steps on the calcite surface (i.e. steps 1 and 2 in Fig. 4b) started to advance, reflecting the growth of a single, presumptively calcian otavite layer (Ot-1). This layer displayed a different lateral deflection signal than that of the calcite surface, in accordance to the results of Hay et al. [29] and is reproduced in the image in Fig. 4b where friction contrast is observed between both surface regions, with the brighter regions corresponding to higher friction. Fig. 4b also shows the formation of four small nuclei on top of Ot-1. These nuclei (Ot-2) represent a second SS layer, displaying higher friction than Ot-1 (Fig. 4c). Once the growing steps in Ot-1 reached the original position of the underlying calcite step (i.e. step 2 reaching the initial position of step 1, marked as 1' in Fig. 4b) their growth rate was reduced from 0.14 ± 0.01 nm/s to 0.03 ± 0.01 nm/s. In other words, the step advancement speed of a layer of calcian otavite growing over a previously formed single layer of calcian otavite is significantly slower than that of calcian otavite growing over calcite. Astilleros et al. [24,25] reported a similar situation for experiments in which MnCO_3 _and (Ca, Sr)CO_3 _were grown on calcite. They referred to this as the "template effect" because the original surface topography was reproduced after a single layer had grown. Higgins and Hu [40] also reported a similar situation with the formation of what was termed a "self-limited" monolayer film on dolomite {10¯1¯4} surfaces.

As more of the original calcite was covered by the calcian otavite, single layer nuclei continued to form preferentially on Ot-1 instead of the calcite surface and additional nucleation took place over previously created islands, resulting in the formation of "stacks" of calcian otavite islands up to 4 or 5 layers. None of these "stacks" showed any higher friction than the double layer of calcian otavite (Ot-2). Repeated experiments (Cd.9 and Cd.12) showed a similar nucleation and growth behavior wherein an initial episode of step advancement was followed by nucleation and growth over previously formed surfaces.

Multilayer growth produced only by step advancement (i.e. no island nucleation) was observed in experiment Cd.7. In this case, a similar situation as that observed for experiment Cd.6 took place, where the advancement speed of the growing layer decreased by more than one order of magnitude after crossing the underlying calcite-calcian otavite boundary. Nevertheless, the growth of additional layers continued uninterrupted and was not affected by the original position of the calcite steps, meaning that calcite substrate control is limited only to the first layer grown over the original crystal.

**Figure 5:**
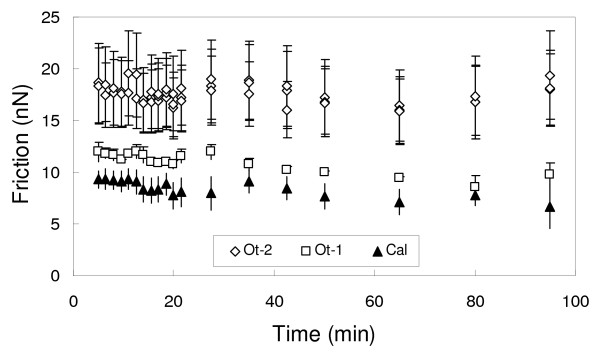
**Friction measurements as a function of time for calcite, Ot-1 and Ot-2 layers for experiment Cd.6**. Error bars correspond to the calculated standard deviation of the friction.

Fig. 5 shows the measured friction as a function of time for the layer of calcite, Ot-1 and Ot-2 from the Cd.6 experiment. This figure reveals that at a load of 25 nN, Ot-1 forms a contact with the AFM tip with a friction force 20% higher than that of the contact with calcite, whereas Ot-2 shows an increase of 80% with respect to the original calcite surface. This is in contrast to the values reported in Fig. 3 where at a load of 25 nN the Cd-rich layer has a higher friction than calcite. Since the experiments where performed with different cantilevers this inconsistency is probably due to a difference in the tip radius. Additionally, the relative differences in friction between calcite, Ot-1 and Ot-2 varied for different experiments performed in Ca-free solutions, even when supersaturation and load conditions were similar, raising additional questions on the effect of other experimental parameters as discussed in the Methods section.

Experiments performed at slightly higher supersaturation, such as experiment Cd.2 (SI_ot _= 3.35), showed a somewhat different friction behavior. In this case no friction difference between the single and double layers of calcian otavite was observed. Also, the advancement speed of the steps growing over the single, initial layer of calcian otavite was not reduced, thus no "template effect" was observed. The step advancement speed measured on the calcite substrate was 0.27 ± 0.03 nm/s in the Cd.2 experiment, significantly higher than that measured in the Cd.6 experiment (0.14 ± 0.01 nm/s) performed at SI_ot _= 2.75. As in the previous experiments, nucleation started only after most of the original calcite surface was already covered by a single layer of calcian otavite, and proceeded at a higher rate than on the experiments performed at lower supersaturations. Nuclei expanded at the same speed as the single calcian otavite layer.

### Film growth from Ca^2+^(aq)-containing inlet solutions

**Figure 6:**
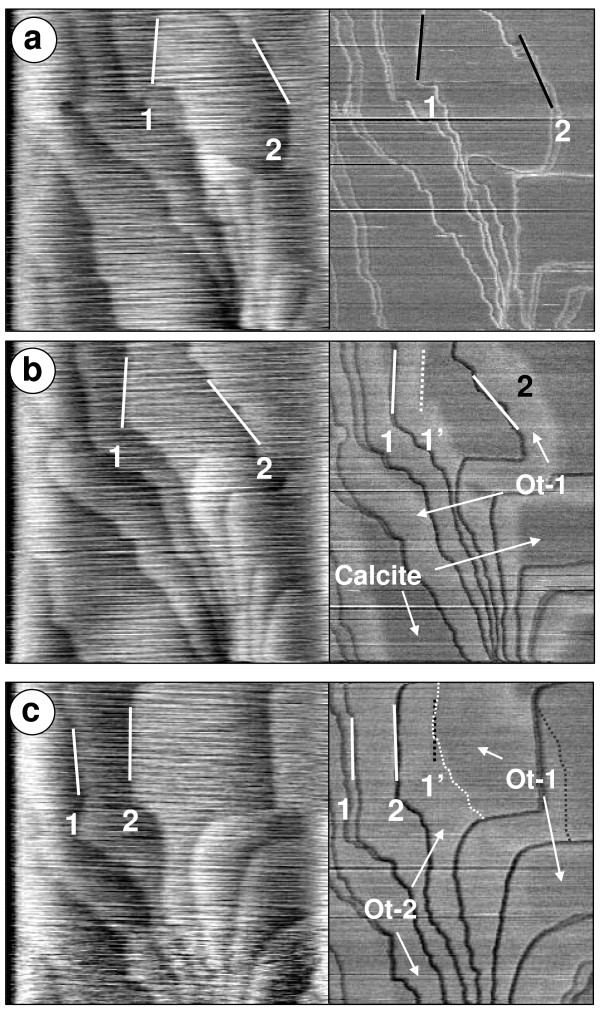
**Height and friction AFM images for experiment Cd.16**. a) calcite surface prior to the introduction of the Ca^2+^(aq)-Cd^2+^(aq)-HCO^-^_3_(aq) solution. The positions of 2 steps (1 and 2) are highlighted. b) 43 min. after introducing the solution, the advancement of steps 1 and 2 is clear in the topography image. In the friction image, the new growth layer (Ot-1) has a higher friction than the original calcite surface. The initial position of step 1 is marked as 1'. c) 80 min. after solution introduction, the further advancement of steps 1 and 2 is marked in the topography image. The friction image shows that step 2 has advanced well-over the initial position of step 1, growing a second layer of solid-solution (Ot-2) which possesses a higher friction than one layer of solid-solution (Ot-1). Image size: 3.3 *μ*m × 3.3 *μ*m.

A similar situation as that illustrated in Fig. 4 was observed when the inlet solutions contained both Cd^2+ ^and Ca^2+^, as can be seen in Fig. 6 which shows a series of AFM images taken during experiment Cd.16. Fig. 6a shows the original calcite surface prior to the introduction of the aqueous solution. Minutes after introducing the solution the steps started to advance indicating the growth of the (Ca, Cd)CO_3 _solid solution, as can be seen by the relative movement of steps 1 and 2. The newly formed, single layer of solid solution (Ot-1) showed higher friction than the original calcite surface. Once the steps reached the underlying contact area between the calcite and the new phase (marked by 1' in Fig. 6b), its advancement speed was reduced considerably as was observed in the experiments performed with the Ca-free solutions. As the steps continued to advance over Ot-1, a contrast in the friction between the double solid solution layer (Ot-2) and Ot-1 was apparent.

**Figure 7:**
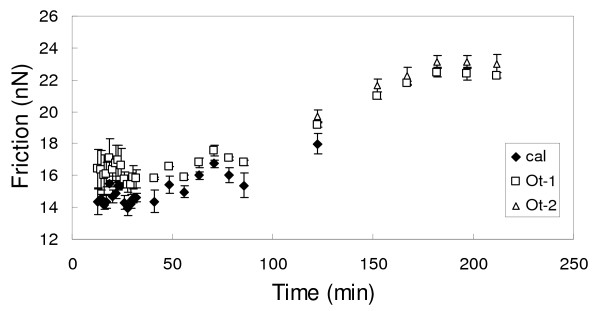
**Friction measurements as a function of time for calcite, Ot-1 and Ot-2 layers in experiment Cd.16**.

Measured values of friction for calcite, Ot-1 and Ot-2 as a function of time are displayed in Fig. 7. The difference in friction between calcite and Ot-1 was small (≈10%) as well as the difference between Ot-1 and Ot-2. Drift in the friction value of the three layers is evident. 100 min after the start of growth, the friction signal measured on all regions increased in the same manner and stabilized after approximately 180 min. The source of drift may have originated from fluctuations in the laser signal arriving at the photodiodes, thereby affecting the load. Regardless of the source of drift, the relative differences in the friction between the different layers remained constant.

## Discussion

### Friction differences between substrate and a single layer of overgrowths

**Figure 8:**
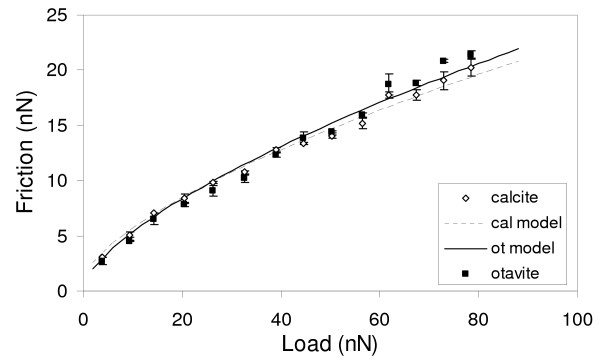
**Load vs friction plot from experiment Cd.1**. Lines represent best-fits performed by using the JKR theory.

As has been pointed out before, Hay et al. [29] associated the change in friction between the calcite substrate and the calcian otavite overgrowths to the change in chemistry, but also suggested that the higher friction in the overgrowths could be due to the strain in this layer. This observation appeared to be backed by the fact that higher lateral force was always observed on the overgrowths compared to that of the calcite substrate. Nevertheless, as has been shown here, there is a reversal of friction contrast at a particular load, hinting to a more complex connection between friction and the overgrowth physical and chemical characteristics. To better describe this relationship, the experimental data from Fig. 3 (experiment Cd.1) was fitted to the JKR model using equations 1, 2 and 4 and displayed in Fig. 8. For the tip-calcite contact, the bulk Young's modulus values for the tip material (Si) and the substrate (calcite) where used in the model, consistent with studies in other systems [47,75]. Values used in the fit were E = 168 GPa [76], *υ *= 0.3 [77] for Si and for calcite, E = 76 GPa, *υ *= 0.32. A tip radius of 40 ± 5 nm was used based on measurements performed after the experiment. The adhesion energy (*γ*) was calculated beforehand by means of equation 3, which relates it to the pull-off force. The pull-off force was obtained by extrapolating the experimental data at low loads (< 25 nN) to zero friction using a JKR type function. Using this approach, an energy of adhesion of 1.7 ± 0.2 mJ/m^2 ^was obtained. Utilizing this value, the full range of experimental data was fitted to a JKR type-curve by adjusting the shear strength, resulting in a value of 0.48 ± 0.05 GPa.

For the calcian otavite contact it was assumed, as a first approximation, that the bulk mechanical properties of otavite applied to a single monolayer of material. Zhang and Reeder [78] reported a bulk modulus for otavite of 97 GPa. This is related to the Young's modulus through the following relation:(9)

where ***K***_***v ***_is the bulk modulus and *υ *the Poisson ratio. No value for the Poisson ratio of otavite was found in the literature, but other carbonates' values are in the range 0.35 – 0.3, which was used to calculate a range of otavite Young's moduli (88–117 GPa). Both values are higher than that reported for calcite (76 GPa) and are in accordance with the reported higher hardness for otavite [79]. Still the question remains on whether these values are representative of a strained monolayer of calcian otavite [80], where the M-O distances will be distorted with regard to their ideal positions. Zhang and Reeder [78] found a linear relation between M-O distances and compressibility of metal carbonates along the c direction. This direction contains a significant vector component parallel to the AFM tip axis, hence it is reasonable to assume that the compressibility of the monolayer will be a value intermediate between that of pure calcite and pure otavite. Therefore a higher value for ***E ***for the monolayer compared to calcite will be expected in this case.

Using the calculated values for ***E ***and following the same approach as in the calcite data, a JKR-type fit was done on the experimental data collected from the Ot-1 film. The calculated adhesion energy is 0.18 ± 0.02 mJ/m^2^, which is an order of magnitude smaller than that found for the calcite contact. Two values of shear strength derived from the fit were 0.57 ± 0.05 and 0.65 ± 0.06 GPa, corresponding to the use of *E *= 88 and 117 GPa, respectively. Although ***E ***and *τ *strongly co-vary in Eqn. 4, the reasonable assumption that a calcian otavite film should have a larger Young's modulus than calcite would lead to *lower film friction at high loads *unless this film forms a contact with larger shear strength (Fig 2b), the latter of which is consistent with the data.

Relatively few fundamental studies have been reported on the factors governing the contact shear strength of single asperity contacts [57]. Results presented here may be explained by considering the energy dissipated at the tip-surface contact due to surface dehydration produced by the scanning tip at high loads, as well as its interaction with adsorbed ions, which has been shown to have an effect on friction [81]. Murdaugh et al. [81] studied the growth of PbSO_4 _and SrSO_4 _films on BaSO_4 _substrates from undersaturated solutions. They explained this behavior by considering the interaction between surface and solute species as defined by the solubility products of the different phases involved. High interaction forces will lead to an increasing adsorption of ions on the substrate surface, creating a local enrichment, and hence an increase in supersaturaion, on the interface, which in-turn will promote growth. Lateral force measurements on the growing films revealed a lower friction than the substrate, which was attributed to lower concentrations and weaker binding of adsorbed ions than those found on the substrate. A similar situation could be expected in the system reported here, since the interactions between CO_3_^2- ^ions and Ca^2+ ^and Cd^2+ ^would be quite different attending to the difference in K_sp _for otavite (10^-12.1^) and calcite (10^-8.48^). In this context the interaction between Cd^2+ ^and CO_3_^2- ^is expected to be stronger than that of Ca^2+ ^and CO_3_^2-^. Therefore a higher concentration of adsorbed CO_3_^2- ^is to be expected over the otavite surface. On the other hand the contribution to friction by tip induced dehydration will lead to higher friction for a material with higher hydration energies. In our case the experimental hydration enthalpies of Ca^2+ ^and Cd^2+^, which are -1659 kJ/mol and -1850 kJ/mol, respectively support the observations at high load where otavite displays a higher friction than calcite. Although the different contributions on friction due to dehydration or interaction with surface species is very difficult to discern, the concentration of water molecules in solution is significantly higher than that of CO_3_^2-^(aq) (55 M vs. 10^-4 ^M respectively). Hence surface dehydration would be expected to contribute more to the total friction on the basis of bulk concentrations.

Results from the data fitting show that friction at low loads is controlled by the adhesion energy so the contacts with lower adhesion energy (calcian otavite) display lower friction. Adhesion has been found to be controlled (at least partially) by electrostatic interactions between the tip and surface [82,83]. These, in turn, will be dependent on solution pH and the pH_PZC _of the tip and substrate. Churchill et al. [84] found that attractive forces in a SiO_2_-calcite contact increased as the solution pH decreased from 9.5, which is the pH_PZC _for calcite. The pH in experiment Cd.1 was 8.4 so a higher adhesion force would be expected in the calcite contact than in that for calcian otavite (assuming similar surface site density) since pH_PZC _= 9 for otavite [85].

### Friction differences between multiple layers of overgrowths

**Figure 9:**
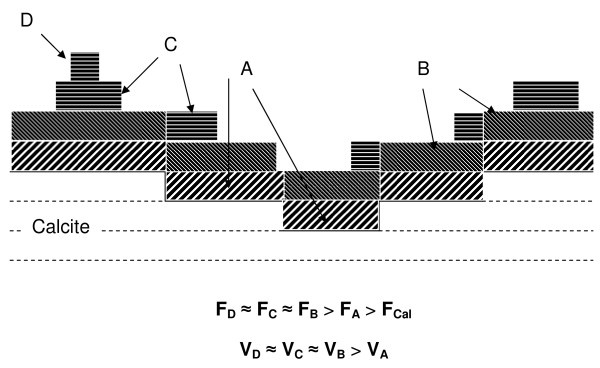
**Representative cross-section of an experiment where growth and friction contrast between layers had been observed**. **F**_**i **_indicates the friction computed for each layer stacking and **V**_**i **_its advancement speed.

Fig. 9 shows a "representative" cross section of a calcite surface following multilayer growth at moderate supersaturation levels (SI = 2–3) on experiments performed with both Ca-rich and Ca-free solutions. Friction of each "stack" was denoted as F_i_, and V_i _refers to its advancement speed. In principle two different scenarios can be envisioned to explain the friction variations between a single and double layer of SS: 1) There is no variation in the composition of the different layers (i.e., an atomically sharp interface) and friction changes between one layer and a double layer reflect only a difference in the mechanical properties due to strain in the overgrowths. 2) There is a variation in the chemistry of the growing layers, which will lead to differing chemical and mechanical properties (the latter possibly strain-related) and thus to a variation in friction.

Previous AFM studies on the growth of solid solutions have usually assumed that the composition of the precipitating layers does not change with depth [24-26,29]. Hay et al [29] found that a double layer of CdCO_3 _was more difficult to dissolve than a single layer, but assumed that this was just due to a relaxation of the epitaxial strain which "stabilized" it and not to a change in composition. Studies have shown, in fact, that strain is relaxed in epitaxially grown layers as film thickness increases [86]. Nevertheless, as discussed in the previous section, the relationship between strain and friction is not straightforward. Furthermore a direct correlation between strain and friction is contradicted by the fact that friction was found to increase from a single layer to a presumed less-strained double layer of overgrowth (Ot-2), and by the fact that no further changes in friction were observed when the formation of additional layers took place. In any case, it is reasonable to expect that a relaxation in the strain of the overgrowths could lead to a change in the mechanical properties as more layers are stacked, resulting in a change in friction. However, experiments performed at the highest supersaturation (Cd.2) showed no change in friction from a single layer to a double layer, even when relaxation must certainly occur. This offers additional evidence against an exclusive correlation between strain and friction.

The second possible explanation for the variation of the friction between a single and a double layer of SS could be that there is a variation in the chemical composition between them. Chiarello et al. monitored the growth of (Ca, Cd)CO_3 _solid solutions [87] and pure otavite [80] over calcite by means of synchrotron X-ray scattering. In [87] they used different initial compositions of growth solutions with different ratios of Ca^2+^/Cd^2+^. However, in each experiment it was reported that the composition of the (Ca, Cd)CO_3 _SS growing over calcite evolved towards the Cd end-member as the film grew thicker. In the case of pure otavite growth [80] they found that the composition of the precipitated layer was pure otavite after a few nm of deposit had formed. In that study however, the resolution was not sufficient to determine a compositional variation between individual layers. Nevertheless, the evidence in Chiarello et al. [87] does demonstrate that the chemical composition of a solid solution film varies as a function of depth as is suggested in the present work and that compositional transitions will be limited to only a few layers.

A mechanism to explain the variation in the chemical composition will necessarily require a degree of control exerted by the substrate on the composition of the precipitating layer. Several authors have investigated systems where this control occurs. For example, Marek et al. [88] found that surface orientation controls the composition of homoepitaxial grown layers of GaAs, whereas Jun et al. [89] found that a carbonate surface can "direct" the form and composition of a precipitating layer of Mn oxide. In terms of minimizing the strain energy in the newly formed phase, growth of a solid solution with a higher Ca content will be favored over that composition predicted from bulk SS-AS thermodynamic considerations. This difference arises because the degree of mismatch between the newly grown film and the calcite substrate will be smaller with higher Ca content, thus reducing the positive increase in Gibbs energy from strain. This tendency was recently demonstrated by [90] in their theoretical calculations on the effect of the composition of Ge_x_Si_1-x _solid solutions grown epitaxially over Si(111) in the transition thickness for a Stranski-Krastanov growth mode [91]. They found that the transition thickness, which is related to the accumulation of strain in the overgrowths, was bigger as the Si content of the solid solution increased. Therefore, the smaller the misfit between the substrate and the Ge_x_Si_1-x _film the higher the relaxation in the strain. Even in the experiments reported herein where Ca-free solutions were introduced, a small amount of Ca^2+ ^would be present due to the dissolution of calcite in the area where the undersaturated (with respect to calcite) jet of solution meets the surface. These Ca^2+ ^ions will be carried away, radially, by the flow into the scan area. The presence of Ca^2+ ^near the surface could lead to Ca^2+ ^incorporation into the film thereby reducing film strain. In light of the above discussion, a pure strain-induced friction contrast mechanism is not supported but the film chemistry has a significant influence on the observed friction.

### Nucleation and growth behavior

The nucleation behavior observed in the experiments performed with Ca-free solutions can be described by the Stranski-Krastanov epitaxial growth mode [91]. This mode is one of the 3 different growth modes established on considerations on the balance between the surface free energy of the substrate, deposit and the interface [92]. The Volmer-Weber mode refers to that produced when the surface free energy of the substrate is smaller than the sum of the interfacial and deposit free energies. In this mode the deposit will growth in the form of 3-dimensional islands to minimize the area covered by the new material. The second mode is called the Stranski-Krastanov mode and occurs when free energy of the substrate is larger than the sum of the interfacial and deposit free energies. In this case the growth takes place initially by a layer by layer mechanism and then is followed by the development of 3-dimensional islands that will minimize the strain accumulated in the deposit. The final growth mode is denoted as Frank-van-der-Merwe and takes place when the substrate free energy equals the sum of the interfacial and deposit surface free energies. This condition is only fulfilled when the growing crystal is the same as the substrate i.e. for homoepitaxial growth. In this case growth takes place by a layer by layer mechanism indefinitely. In the present study, the first 1–2 layers of calcian-otavite solid solution growth epitaxially over calcite, but strain in those layers drives a mechanism for strain-relief that includes compositional variations and/or defect formation. The local strain relaxation leads to repetitive nucleation on localized regions, forming multilayer, pseudo 3-D nuclei.

In contrast to the high supersaturation experiments, for those performed at low supersaturation it was shown that the measured step advancement speed of the second layer of SS decreased considerably as compared to that measured on a single SS layer. The reduction of step velocity for a second layer of SS has been observed in the growth of different metal carbonate solid solutions over calcite [24-26,28]. Astilleros et al. [26] proposed that the step advancement reduction was due to a relaxation in the strain produced by the growth of a second layer of solid solution. In their model, the composition of the different layers would be the same but the first layer will incorporate the "impurities" randomly, whereas in subsequent layers it will be directed by the substrate. This, in turn would lead to a progressive reduction of the step advancement until it stopped, plus an inhibition of nucleation. Contrary to what Astilleros et al. [26] postulate, in the present work no further reduction of the step advancement speed was observed, not even after 5 or 6 layers had grown. In these experiments the only observed step speed reduction occurred between the first and second layer of solid solution. The step speed reduction effect can be explained in light of the slower growth kinetics of otavite with respect to pure calcite and the fact that the first layer grown has an intermediate composition between the two end members.

### The role of supersaturation

Regarding the experiments performed with Cd-rich solutions, supersaturation seems to play an important role not only in the growth process [11], but also in the friction differences observed. It has been noted that supersaturation may influence the composition of the solid [11]. This could explain why at higher supersaturation no contrast between the monolayer and the double layer is observed. Also a higher degree of supersaturation promotes a faster growth rate of the initial monolayer (effectively doubling itself from a SI of 2.75 to 3.35), further impeding any possible dissolution of the calcite surface and thereby reducing the availability of Ca^2+ ^in the surrounding fluid. A higher degree of supersaturation will also drive the system to a higher incorporation of Cd^2+ ^in the precipitating layer. Under these conditions, the composition of the initial single layer will probably be equal to that of subsequent layers producing little or no contrast in friction between film layers and no template effect.

The significant geochemical implications of the above observations stem from the need to better predict how minor and trace element composition in minerals is related to the fluid composition from which mineralization took place. While equilibrium thermodynamic principles allow prediction of limiting compositions in systems that rapidly approach equilibrium, reactions of solid solutions with aqueous fluids may not easily achieve an equilibrium state. In the latter case, bulk thermodynamics cannot be used to predict surface or near-surface composition of minerals and therefore, kinetic factors must be incorporated into geochemical models. Identifying and quantifying the rate-controlling mechanisms requires further investigations in which surface and fluid composition are correlated, preferably with spatial resolution sufficient to resolve heterogeneities that may exist at the nanometer scale. These studies, while performed on only one relatively simple mineral-water interface system, demonstrate that LFM may contribute important chemical information with high spatial resolution through its sensitivity to surface ion hydration enthalpy and surface charge, and lead to important developments in geochemical modeling of the mineral-water interface.

## Summary

Results presented in this paper corroborate those from previous lateral force studies in that LFM can provide additional useful information to recognize compositional contrast in mineralization studies. This work demonstrated that previous descriptions of friction differences between substrates and films as directed solely by differences in strain may not be universal. Specifically, the observations of friction contrast reversal, arising from differences in the load dependence of friction for calcian-otavite films and calcite, demonstrated a need for additional chemical and mechanical considerations beyond a simple friction-strain relationship. This load dependence was further explored by fitting successfully the experimental data using the JKR continuum mechanics theory for single asperity contacts. These studies showed that higher friction for the calcite-tip contact at low loads was due to significant differences in adhesion between the tip and the film and calcite substrate. At higher loads, the higher friction of the calcian otavite-tip contact indicated larger shear strength associated with this contact. The contact shear strength could correlate with dehydration enthalpy that must be overcome by the scanning tip. Hence, the higher friction of the calcian otavite contact will be related to the higher hydration enthalpy of Cd^2+^. Differences in adhesion at low loads are probably governed by electrostatic interaction between tip and sample and therefore related to the differences in the pH_PZC _of the film and calcite substrate.

The observed friction contrast between a single and a double layer of solid solution suggested a difference in composition between layers. Compositional evolution during multilayer film growth reduces strain in the overgrowth by decreasing misfit with the underlayer. The change in layer composition with film depth requires a degree of control by the substrate on the composition of the overgrowths, a process that is not fully understood at this point. As shown in experiments at higher supersaturation where friction was similar on all film layers, the control of the substrate on film composition may be overcome by the higher driving force for film formation. Additional studies using surface sensitive chemical analytical techniques will be required to fully understand this phenomenon, but nanometer-scale friction measurements clearly provide important surface characteristics toward further development of knowledge in the growth of more chemically complex minerals. To facilitate the role of LFM in mineral surface chemistry investigations, future efforts should emphasize inclusion of experimental variables such as tip size and shape to permit multiple experiment comparisons.

## Competing interests

The authors declare that they have no competing interests.

## Authors' contributions

PC performed the AFM and LFM measurements, contributed to the design of experiments, fitting of data and drafted the manuscript. SH contributed to the original concepts for investigation, contributed to the experimental design and manuscript drafts and provided suggestions for the data fitting. Both authors read and approved the final manuscript.
